# NONCOMMUNICABLE DISEASES AND HEALTH CARE UTILIZATION: A STUDY OF OLDER ADULTS IN INDIA

**DOI:** 10.1093/geroni/igad104.2427

**Published:** 2023-12-21

## Abstract

Non-communicable diseases (NCDs) have become one of the leading causes of death in India, and it’s more prevalent among aged population. Access to healthcare facilities (HCFs) can reduce this mortality burden considerably. This study addressed the rural-urban disparity in accessing HCFs for the treatment of NCDs among older adults. Using data from LASI wave-1 (2017-18), 10,865 older adults (aged 60+) who accessed HCFs for NCD treatment were analyzed. Bivariate and multivariate analysis was applied to calculate the distance to avail HCFs through public and private transportation for the last outpatient visit. The findings indicated that NCD patients highly preferred to visit private HCFs for their treatment in India. Sixty-eight percent of the rural NCD patients residing up-to 5 kilometres (km) distance from HCFs were utilizing private transport, whereas it was around four-fifths of urban NCD patients. As the distance to HCFs increases from 5km and more, the use of public transportation by NCD patients in rural areas were significantly higher compared to their urban counterparts. However, private transport use for HCFs located between 5-to-50km was not varied much by place of residence. Findings showed that there was no significant variation in health expenditure at public HCFs among rural-urban patients. Nearly one-fourth of urban and one-fifth of rural older adults were utilizing public HCFs free-of-cost. Urban residents who visited private HCFs had to spend eight times more than those who opted for public HCFs. Only nineteen percentage older adults have health insurance coverage in India, indicating considerable burden of out-of-pocket expenditure.

